# Chemoprophylaxis to Prevent Deep Venous Thrombosis in Patients Hospitalized for Pancreatitis: Beneficial or Harmful?

**DOI:** 10.7759/cureus.19645

**Published:** 2021-11-16

**Authors:** Raja S Vadlamudi, Venkata Vinod Kumar Matli, Viveksandeep Thoguluva Chandrasekar, Aditya Kalakonda, Sekou R Rawlins

**Affiliations:** 1 Gastroenterology, State University of New York Upstate Medical University, Syracuse, USA; 2 Internal Medicine, State University of New York Upstate Medical University, Syracuse, USA; 3 Gastroenterology, Augusta University Medical College of Georgia, Augusta, USA; 4 Gastroenterology, St Elizabeth Physicians, University of Kentucky, Crestview Hills, USA

**Keywords:** acute necrotizing pancreatitis, splanchnic venous thrombosis, pancreatitis, dvt prophylaxis, deep vein thrombosis (dvt), peri-pancreatic hemorrhage, splenic vein thrombosis, hemorrhagic pancreatitis

## Abstract

Introduction: Vascular complications in pancreatitis generally occur in the form of hemorrhage or thrombosis. Pancreatitis resulting in splanchnic thrombosis has been well studied, but the cause of this correlation has not been studied in the current era of increasing anticoagulant use for deep venous thrombosis (DVT) prophylaxis. Hemorrhagic pancreatitis and peri-pancreatic bleeding are also known phenomena encountered in relation to pancreatitis, but these risks are not well established in the setting of chemical prophylaxis for DVT.

Objectives: Our objective was to identify whether chemical DVT prophylaxis in pancreatitis harms the patient by increasing the risk of hemorrhagic conversion of pancreatitis or peri-pancreatic hemorrhage or if it is beneficial by preventing splanchnic venous thrombosis in the abdominal vasculature that surrounds the pancreas.

Methods: We undertook a retrospective chart review with approval from the Institutional Review Board on patients who were hospitalized for or developed pancreatitis during their hospital stay from April 2014 to July 2015. We reviewed the charts for imaging suggestive of venous thrombosis or the development of intra-abdominal hemorrhage at admission during hospitalization and within 30 days after hospitalization. We also reviewed the methods of DVT prophylaxis to identify any correlation with the risk of hemorrhage or thrombosis. A bedside index of severity in acute pancreatitis score was used within 24 hours of admission to calculate the severity of the patients’ pancreatitis. The data collected were analyzed for descriptive statistics, correlation using Pearson’s coefficient, and multivariate regression analysis using Microsoft Excel and SPSS Inc. Released 2009. PASW Statistics for Windows, Version 18.0. Chicago: SPSS Inc.

Results: This study included 389 patients who met the inclusion criteria. Of these, 74.6% of patients received chemical prophylaxis, mostly low molecular weight heparin, and 18.5% of patients were not on chemical or mechanical means of DVT prophylaxis. Only 12 patients (3%) had complications related to thrombosis and hemorrhage. Seven patients had splanchnic venous thrombosis, one had a hemorrhagic conversion of pancreatitis, three had a peri-pancreatic hemorrhage, and one had both the hemorrhagic conversion of pancreatitis and peri-pancreatic hemorrhage. Ten patients out of 12 patients had complications before admission, and nine of the 12 patients were on chemical prophylaxis. Pearson’s coefficient showed no statistically significant correlation between the incidence of complications and the use of chemical DVT prophylaxis. Multivariate analysis showed no specific variable that increased the risk of complications.

Conclusions: Our study showed that chemoprophylaxis for DVT in patients hospitalized for acute pancreatitis is neither harmful by causing hemorrhagic conversion of pancreatitis, peri-pancreatic hemorrhage nor beneficial by preventing splanchnic venous thrombosis.

## Introduction

Acute pancreatitis is an inflammatory process that can cause local and systemic pancreatic and peripancreatic complications, including organ failure. This is the most common gastrointestinal reason for hospitalization in the US [1].

The annual incidence of acute pancreatitis in the US is approximately 34 per 100,000 people, and this has increased over the last decade because of a marked increase in obesity rates and incidence of gallstones [2]. 

Complications of pancreatitis can range from systemic inflammatory response syndrome and organ failure, which usually occur in the first week of the disease process, to peripancreatic fluid collection, including peripancreatic hemorrhagic conversion, pancreatic and peripancreatic necrosis, and peripancreatic vascular complications comprising splanchnic venous thrombosis. 

Splanchnic venous thrombosis, which includes the splenic, portal, and/or superior mesenteric veins, is seen on imaging studies in patients with acute pancreatitis with an approximate incidence rate of 1%-24% [3-6]. Management of acute pancreatitis may cause spontaneous clearance of thrombosis. If there is any suspicion of potential risk for liver decompensation or an ensuing compromise in intestinal perfusion, therapeutic anticoagulation is indicated despite concern for hemorrhage.

Given the propensity of complications associated with pancreatitis, we decided to study the impact of deep vein thrombosis (DVT) prophylaxis on patient outcomes. No other studies have been conducted to identify the clinical courses of similar patients. We aimed to retrospectively analyze the clinical course of pancreatitis in a subset of hospitalized patients receiving DVT prophylaxis versus those who did not receive anticoagulation or mechanical modes of venous thromboembolism (VTE) prophylaxis.

Complications in the current era of increasing anticoagulant use for DVT prophylaxis have not been well studied, and there are wide lacunae in the research that draw our attention to this study. 

## Materials and methods

Methods

Design

This study comprised of retrospective chart reviews. Data were collected from patients admitted to our hospital between April 1^st^, 2014, to July 31^st^, 2015.

Target Population

All patients who were hospitalized for or had developed pancreatitis during their hospital stay between April 2014 and July 2015 were included. The patient list was obtained from the medical record database (EPIC) using ICD codes 577.0 (acute pancreatitis), 577.1 (chronic pancreatitis), 072.3 (mumps pancreatitis), and 095.8 (syphilitic pancreatitis). The above diagnoses included acute-on-chronic pancreatitis.

Inclusion Criteria

Patients admitted from April 1^st^, 2014, to July 31^st^, 2015, with recorded diagnoses of pancreatitis using ICD codes (as noted above) were included in this study. Pancreatitis, including type based on acuity, was confirmed based on the following: Acute pancreatitis, the patients had to meet two of the following three criteria; (i) abdominal pain consistent with the disease, (ii) serum amylase and/or lipase greater than three times the upper limit of normal, and/or (iii) characteristic findings from abdominal imaging; acute on chronic; or chronic, presenting as a chronic, inflammatory process of the pancreas, characterized by irreversible morphologic changes noted on radiological (CT/MRI/US) or endoscopic (EUS/ERCP) work-up.

Exclusion Criteria

Patients who did not meet the inclusion criteria were excluded from the study.

Data Collection and Analysis

We reviewed the charts of patients diagnosed with pancreatitis with a focus on images that were suggestive of venous thrombosis or the development of intra-abdominal hemorrhage during and 30 days after hospitalization. We also reviewed the method of DVT prophylaxis and correlated it with patient outcomes in the setting of pancreatitis and tried to identify a temporal relationship between the risks of hemorrhage and venous thrombosis in patients who either did not receive DVT prophylaxis or did so routinely. If the same patient was admitted multiple times for pancreatitis, each admission was treated as a different entity during our analysis if readmitted after 30 days since the previous admission.

The bedside index of severity in acute pancreatitis (BISAP) score was used to calculate the severity of pancreatitis within 24 hours of evaluation of the patient.

The data collected were analyzed for descriptive statistics, correlation using Pearson’s coefficient, and multivariate regression analysis using Microsoft Excel and SPSS Inc. Released 2009. PASW Statistics for Windows, Version 18.0. Chicago: SPSS Inc. This was used to test the hypothesis that chemical DVT prophylaxis decreased the formation of microthrombi, which are associated with the worsening of pancreatitis and its systemic manifestations.

## Results

Patients were assigned one point for each of the following during the first 24 hours: BUN >25 mg/dL, impaired mental status, systematic inflammatory response syndrome (SIRS) using the same criteria as the SIRS score (Table 1), age >60 years, or the presence of pleural effusion. Patients with a score of zero had a mortality rate of less than 1%, whereas patients with a score of five had a mortality rate of 22%. Points between 0-2 indicate lower mortality (<2%), and scores between 3-5 represent higher mortality (>15%).

**Table 1:** Systemic Inflammatory Response Syndrome SIRS: Systemic Inflammatory Syndrome. BPM: beats per minute; pCO2: partial pressure of carbon dioxide; WBC: white cell count.

SIRS Criteria [7]	
Conditions	Criteria met if two or more of the following have these criteria present.
Temperature	< 36 °C or > 38 °C
Heart rate	> 90 bpm
Tachypnea, or an arterial pCO2	> 20 breaths per minute < 32 mmHg
WBC	< 4000 cells/mm³ (4 × 109 cells/L) or > 12,000 cells/mm³ (12 × 109 cells/L), or > 10 % immature neutrophils (left shift)

The medical records of 463 patients with a recorded diagnosis of pancreatitis were reviewed. Of these, 74 (16%) patients were excluded because they did not meet the inclusion criteria, mainly based on the definition of pancreatitis. The median age at diagnosis was 49 years, ranging from 2 to 97 years. Females (205, 52.7%) were slightly more common than male patients (184, 47.3%). The median length of stay (LOS) was four days, with a range extending from one to 168 days. The LOS of 168 days was an outlier, and if ignored, the range was from one to 61 days (Table 2). The patient with a LOS of 168 had acute pancreatitis with a BISAP score of one at the time of admission. This patient later developed necrosis and acute abdominal compartment syndrome requiring surgery, followed by complications related to surgery but not due to pancreatitis. The patient did not have any thrombotic or hemorrhagic complications related to pancreatitis.

**Table 2:** Demographics and characteristics of the study population

Demographics and Characteristics of the study population	
Total patient records reviewed	463
Patients included	389 (84%)
Patients excluded	74 (16%)
Age at diagnosis	Years
Median	49
Range	2-97
Sex	N (% included patients)
Male	184 (47.3%)
Female	205 (52.7%)
Type of Pancreatitis	N (% included patients)
Acute	299 (76.9%)
Acute on chronic	78 (20.1%)
Chronic	12 (3.1%)
Bedside index of severity in acute pancreatitis score	N (% of included patients)
Low risk of mortality (0-2 score)	353 (90.7%)
High risk of mortality (3-5 score)	36 (9.3%)
Length of Stay	Days
Median	4
Range	1-61
Deep Venous Thrombosis Prophylaxis	
Chemical	290 (74.6%)
Low molecular weight heparin	164 (42.2%)
Heparin	117 (30.1%)
Therapeutic anticoagulation (Warfarin)	9 (2.3%)
Mechanical	27 (6.9)
None	72 (18.5%)
Patients on Anti-platelet therapy	
Aspirin	54 (14.4%)
Plavix	3 (0.8%)

Acute pancreatitis was the most frequent type (299, 76.9%), followed by acute on chronic (78, 20.1%) and then chronic pancreatitis (12, 3.1%). The average BISAP score was one with a range of zero to five. Most of the patients had a low risk of mortality (353, 90.7%) based on a BISAP score of ≤2.

In our analysis, 290, 74.6% of patients received chemical prophylaxis, mostly with LMWH followed by regular heparin, and had already been on anticoagulation for other thrombotic/prothrombotic conditions. Although there is a great deal of awareness regarding the initiation of DVT prophylaxis, approximately 18.5% of patients were not on chemical or mechanical DVT prophylaxis. Most of these patients were in the pediatric age group (≤ 18 years). Only 14.4% of patients received aspirin or antiplatelet agents during hospitalization.

Of the 389 included patients, only 12 (3%) had complications; 1.8% had complications related to thrombosis, and 1.3% related to hemorrhage or hemorrhagic conversion. Seven patients had splanchnic venous thrombosis, one had a hemorrhagic conversion of pancreatitis, three had a peripancreatic hemorrhage, and one had both the hemorrhagic conversion of pancreatitis and peripancreatic hemorrhage. In 10 of the patients, the complications were present at the time of admission. One had splenic venous thrombosis after 25 days (receiving chemical prophylaxis with regular heparin), and one had peripancreatic hemorrhage approximately 30 days after hospitalization (receiving chemical prophylaxis with LMWH). Nine out of 12 patients received chemical DVT prophylaxis either with LMWH or regular heparin, and the remaining three patients received mechanical DVT prophylaxis or no prophylaxis. Seven of the 12 patients with complications had a low BISAP score (≤2), and the remaining five patients had a BISAP score of three. None of these patients had a very high BISAP score of ≥ 4.

Most of these complications (3%) occurred before hospitalization. However, if we consider them as having occurred during hospitalization while on anticoagulation, analysis with Pearson coefficient still shows no statistically significant correlation between the incidence of complications and the use of chemical DVT prophylaxis (Table 3).

**Table 3:** Correlations between chemical DVT prophylaxis, splenic vein thrombosis, hemorrhagic conversion of pancreatitis, peripancreatic hemorrhage. SVT: Splenic vein thrombosis, HCP: hemorrhagic conversion of pancreatitis, PPH: Peripancreatic hemorrhage, DVT: Deep venous thrombosis. Y: Yes, N: No. * Correlation is significant at the 0.05 level (2-tailed). ** Correlation is significant at the 0.01 level (2-tailed).

Correlations between chemical DVT prophylaxis, splenic vein thrombosis, hemorrhagic conversion of pancreatitis, peripancreatic hemorrhage.				
Correlations	Chemical DVT prophylaxis	SVT Y/N	HCP Y/N	PPH Y/N
Chemical DVT prophylaxis Pearson Correlation	1	0.035	-0.123*	0.001
Sig. (2-tailed)		0.495	0.015	0.983
N	389	389	389	389
SVT Pearson Correlation	0.035	1	-0.010	-0.014
Sig. (2-tailed)	0.495		0.848	0.786
N	389	389	389	389
HCP Pearson Correlation	-0.123*	-0.010	1	0.349**
Sig. (2-tailed)	0.015	0.848		
N	389	389	389	389

A multivariate analysis (logistic regression) showed no specific variable that may increase the risk of complications (Table 4), except for the relationship between the presence of pleural effusion and peripancreatic hemorrhage.

**Table 4:** Study between different variables and SVT, HCP and PPH SVT: Splenic vein thrombosis, HCP: Hemorrhagic conversion of pancreatitis, PPH: Peri-pancreatic hemorrhage, DVT: Deep venous thrombosis, BUN: Blood Urea Nitrogen, AMS: Altered Mental Status, SIRS: Systemic Inflammatory Response Syndrome

Study between different variables and SVT, HCP and PPH						
Coefficient:Dependent Variable: SVT Y or N						
Model		Unstandardized Coefficients		Standardized Coefficients	t	Sig.
		B	Std. Error	Beta		
1	(Constant)	.009	.022		.404	.687
	Age at Diagnosis (years)	-.001	.001	-.094	-1.132	.258
	Length of Stay (days)	.001	.001	.126	2.342	.020
	Chemical DVT Prophylaxis	.014	.017	.046	.818	.414
	BUN (1 or 0)	.046	.021	.124	2.219	.027
	AMS (1 or 0)	.011	.037	.016	.305	.761
	SIRS (1 or 0)	.008	.015	.030	.556	.578
	Age 60 (1 or 0)	.030	.024	.101	1.277	.202
	Pleural effusion (1 or 0)	.000	.031	.001	.016	.988
Coefficient: Dependent Variable: HCP Y or N						
1	(Constant)	.000	.012		-.024	.981
	Age at Diagnosis (years)	.001	.000	.149	1.786	.075
	Length of Stay (days)	.000	.000	-.018	-.329	.743
	Chemical DVT Prophylaxis	-.029	.009	-.177	-3.149	.002
	BUN (1 or 0)	.008	.011	.041	.739	.460
	AMS (1 or 0)	-.009	.020	-.024	-.453	.651
	SIRS (1 or 0)	.004	.008	.024	.453	.651
	Age 60 (1 or 0)	.001	.013	.006	.075	.940
	Pleural effusion (1 or 0)	-.010	.017	-.032	-.615	.539
Coefficients: Dependent Variable: PPH Y or N						
1	(Constant)	-.001	.017		-.077	.939
	Age at Diagnosis (years)	.000	.000	.028	.335	.738
	Length of Stay (days)	.000	.000	-.024	-.451	.652
	Chemical DVT prophylaxis	-.001	.013	-.006	-.111	.912
	BUN (1 or 0)	.017	.016	.060	1.071	.285
	AMS (1 or 0)	.042	.028	.079	1.480	.140
	SIRS (1 or 0)	.005	.011	.026	.479	.632
	Age 60 (1 or 0)	-.013	.018	-.055	-.701	.484
	Pleural effusion (1 or 0)	.090	.024	.198	3.796	.000

Pearson’s coefficient analysis showed no statistically significant correlation between LOS and chemical DVT prophylaxis (Table 5).

**Table 5:** Correlation between patient’s length of stay and chemoprophylaxis for DVT DVT: Deep venous thrombosis

Correlation between patient’s length of stay and chemical prophylaxis for DVT		
Correlation	Length of stay (Days)	Chemical DVT prophylaxis
Length of stay Pearson Correlation	1	0.050
Sig. (2-tailed)		0.322
N	389	389

## Discussion

DVT is a phenomenon often encountered during hospitalization, and several measures are being taken to prevent this, given the associated increased risk of morbidity. Hospitalized patients are at an increased risk of DVT due to prolonged periods of immobility. In recent years, DVT prophylaxis with anticoagulants, such as regular heparin and low-molecular-weight heparin, has been ordered reflexively for all admitted patients, as this strategy does not have any frank contraindications [8]. Moreover, patients with acute pancreatitis are at unusually high risk for extremity DVT. Umapathy et al. showed that 1% of acute pancreatitis patients are associated with extremity DVT, likely secondary to prolonged hospitalization and an inflammatory cytokine cascade. This emphasizes the need for DVT prophylaxis and ambulation in patients with acute pancreatitis [9]. Additionally, Chung et al. conducted a nationwide retrospective analysis of a cohort of patients with acute pancreatitis for the development of VTE. They showed that this cohort is at a higher risk for VTE than the control patients, irrespective of age, sex, or comorbidities. The patients showed a 1.86-fold higher adjusted hazard ratio (aHR) of DVT and a 1.92-fold higher aHR of pulmonary embolism than did the controls [10].

Maatman et al. found that patients with necrotizing pancreatitis (NP) had the highest incidence rate of VTE (57%) in any hospitalized patients. Further, this study showed that the regular chemical DVT prophylactic dose is inadequate to prevent the development of DVT and is likely the reason for the higher incidence rates in NP patients [11].

Splanchnic venous thrombosis, which includes the splenic, portal, and/or superior mesenteric veins, is seen on imaging studies in patients with acute pancreatitis with an approximate incidence rate of 1%-24% [3-6]. Management of acute pancreatitis may cause spontaneous clearance of thrombosis. If there is any suspicion of potential risk for liver decompensation or an ensuing compromise in intestinal perfusion, therapeutic anticoagulation is indicated despite concern for hemorrhage [12].

Thrombotic complications have been known to be more common in alcohol-induced, necrotizing, and chronic pancreatitis. The pathogenesis of venous thrombosis has been suggested to involve stasis, spasm, and mass effects from the surrounding inflamed pancreas and direct damage to the venous wall by liberated enzymes [5]. Although the pathogenesis of splanchnic venous thrombosis is unclear, complications such as portal hypertension, liver decompensation, and intestinal ischemia have been reported [5].

Venous thrombosis of the splenic vein is more common than in the superior mesenteric or portal veins [4]. The high variability in the incidence rate (1%-24%) is because of the severity of acute pancreatitis and also due to differences in imaging modalities and technical expertise. Ultrasound imaging is difficult in some patients, such as those who are obese or with abdominal gas or in whom splenic venous thrombosis may easily occur. To reduce this high variability, three-dimensional computed tomography or magnetic resonance imaging may be required to confirm the diagnosis of splenic vein thrombosis. Assessment of the exact incidence rate will be useful when initiating therapeutic anticoagulation when the diagnosis of splenic vein thrombosis is confirmed [13]. 

Splenic vein thrombosis in chronic pancreatitis is estimated to be up to 12% due to its anatomic location along the posterior surface of the pancreas [14].

Our study showed a splanchnic venous thrombosis occurrence rate of 1.8% in patients hospitalized for pancreatitis. A study by Harris et al. reviewed 2,454 patients admitted with a diagnosis of acute pancreatitis over 10 years and revealed splanchnic venous thrombosis in 45 patients; this is also an incidence rate of approximately 1.8% [15]. 

Hemorrhagic pancreatitis, or peripancreatic hemorrhage, is one of the most life-threatening complications of pancreatitis. It is usually due to erosion of a major pancreatic or peripancreatic blood vessel, or the formation and subsequent rupture of an arterial pseudoaneurysm. Our study showed that hemorrhagic complications associated with pancreatitis occur at a low frequency of approximately 1.3%; however, in the past, it has been reported to vary between 1.2% and 14.5% [3]. Our study did not show any correlation between higher BiSAP scores and the risk of thrombotic or hemorrhagic complications from pancreatitis. The pathogenesis of hemorrhagic complications is multifactorial. One of the factors is mediated by severe pancreatic inflammation and pancreatic necrosis, which occurs during the early phase of acute pancreatitis. Local spread of the inflammatory process and extravasation of proteolytic and lipolytic enzymes further exacerbate the necrotizing process and initiate damage to any proximal vascular structure, rendering them susceptible to subsequent pseudoaneurysm formation or rupture [16]. Hemorrhage tends to occur more frequently in patients requiring surgery for necrosectomy and debridement of infected pancreatic sequestra (sequestrectomy) [3].

## Conclusions

The increased risk of DVT in hospitalized patients has been demonstrated in many studies. Currently, there is often a knee-jerk reaction of the admitting physician to keep every hospitalized patient on DVT prophylaxis, with the majority receiving chemical prophylaxis using heparin products. Patients admitted for pancreatitis are at a critical crossroads with an increased risk of thrombotic and hemorrhagic complications related to pancreatitis, along with an established increased risk for DVT from poor mobility and inflammatory cytokine cascade itself in most of the patients. Our study showed that chemoprophylaxis for DVT in patients hospitalized for acute pancreatitis is neither harmful by causing hemorrhagic conversion of pancreatitis, peri-pancreatic hemorrhage nor beneficial by preventing splanchnic venous thrombosis. Our results indicate that chemical DVT prophylaxis is not contraindicated in patients admitted for any type of pancreatitis, including those with major complications.
